# How gender-specific are predictors of post-MI HRQoL? A longitudinal study

**DOI:** 10.1186/s12955-020-01439-4

**Published:** 2020-06-26

**Authors:** Dorota Wlodarczyk, Urszula Zietalewicz

**Affiliations:** Department of Medical Psychology and Medical Communication, Medical University of Warsaw, Warsaw, Poland

## Abstract

**Purpose:**

Extant research shows that health-related quality of life (HRQoL) differs between female and male survivors of myocardial infarction (MI), but the reasons for this are not fully understood. We aimed to examine the predictors of HRQoL in female and male survivors during the first year after MI.

**Methods:**

At timepoints 1 and 2, the sample comprised 222 MI survivors (59 women and 163 men; mean age 53.84 years, range 24–65) referred for in-patient cardiac rehabilitation. This number dropped to 140 participants (42 women and 98 men) at the third timepoint, approximately one year after the MI. We examined the gender differences in various predictors of physical and mental HRQoL: demographic factors (e.g., age, education, marital status), disease-related factors (pre- and post-MI), personality and coping with stress.

**Results:**

Initially, both physical and mental HRQoL were lower in women than men, but the differences disappeared at timepoint 3. Stepwise regressions performed separately for men and women revealed that the factors shaping HRQoL were different in both genders; they also changed over time. Substantially fewer factors predicted physical HRQoL in women than in men. Trait anxiety seems to play a similarly negative role in both genders.

**Conclusions:**

The psychosocial resources that influence HRQoL were different for women and men. There were also differences concerning predictors of HRQoL dimensions. Further studies with a different or broader range of predictors are needed, especially among women.

## Introduction

Health-related quality of life (HRQoL) is a valid indicator of cardiac patients’ adaptation to their condition and the effectiveness of their treatment. It is a multidimensional concept encompassing the physical, emotional and social components of the effect of illness and treatment, as perceived by the patient [1, 2]. A systematic review [3] revealed that the most commonly cited HRQoL model was that of Wilson and Cleary [4], which consists of five hierarchical levels linking biological functions with symptoms, functional status, perception of general health and overall quality of life.

Kang et al. [5] posited that the correlates of HRQoL in MI patients could be organised into four categories: demographic, disease-related, behavioural and psychosocial (ability to cope with stress and personality). They identified the limited number and scope of HRQoL predictors included in single studies as the main weakness of the research they reviewed.

### Gender differences in HRQoL

A number of studies indicate that HRQoL is gender-dependent. There is some evidence that after an MI, women report lower HRQoL than their male counterparts. Most of the differences were observed at short- and medium-term follow-up (up to 3 years after MI), and among patients less than 70 years old; after longer periods (2.5 to 10 years after MI) and among older patients (> 70 years) the gender difference disappeared [6–8]. This difference in HRQoL is dangerous and disadvantageous for women, as low HRQoL and lack of improvement in HRQoL have been shown to predict a recurrence of cardiovascular events 6 [9] and 13 years post-MI [10].

There are questions about the causes of gender differences in HRQoL and the potential gender specificity of its predictors in MI survivors. Identifying the factors responsible for women’s low HRQoL would make it possible to develop approaches to healthcare that could improve outcomes for female patients [9]. Gender differences in HRQoL determinants may be partly due to gender differences in the course and treatment of MI. Angina is more prevalent in women than men but is treated less aggressively [11]. In general population samples, women score lower than men on the majority of scales measuring the quality of life [8], which has prompted researchers to look outside the clinic for the causes of gender differences in HRQoL after MI.

Pettersen et al. [8] showed that in women, the physical HRQoL outcomes were negatively associated with demographic factors and comorbidities, whereas in men they were more strongly associated with characteristics and consequences of MI. In women, none of the variables investigated were associated with mental HRQoL, whilst in men, HRQoL was negatively associated with education, known MI site and smoking. Gijsberts et al. [12] concluded that in women, HRQoL is determined by menopausal status [13] and psychosocial factors, rather than by cardiac risk factors, cardiac history or other general patient characteristics. Leung Yinko et al. [14] showed that even after adjusting for clinical characteristics and treatment, gender-related factors such as femininity score, social support and responsibility for housework predicted HRQoL in younger patients (< 55 years) with acute coronary syndrome. It seems that women’s psychosocial resources may not be sufficient to cope with MI when they are also burdened with heavy occupational and household responsibilities, and this may affect how they perceive and cope with cardiac events [15].

The potential effects of psychosocial factors in predicting HRQoL is understudied. Among them, personality traits such as type D personality [16] or anxiety and anger/hostility traits [17] decrease HRQoL in different groups of cardiac patients. As a counterpoint, beneficial effects from a sense of coherence [16] or dispositional optimism [18] have been also observed, however, it is unclear whether these effects vary by gender. Similarly, the results of the research on gender differences in associations of coping with HRQoL after MI are mixed [19–21]. In this context, the effects of trait curiosity, defined as a positive attitude towards novelty and openness to experiences [22], can be especially important.

### Aim of the study

The existing research on the determinants of HRQoL in females and males after cardiac events only partially explains the observed discrepancies. To get a fuller insight into the problem, we investigated factors from all the main categories of determinants: sociodemographic, clinical and psychosocial. The impact of these determinants changes depending on the stage of recovery from the cardiac event [17], and so this was also included in the study. Due to the exploratory character of our study, we focused on the examination of HRQoL predictors separately in female and male MI survivors at three different timepoints during the first year after MI: at the beginning and the end of cardiac rehabilitation, and one year after its completion. We also aimed to compare the levels of HRQoL and its determinants in females and males.

## Materials and methods

### Procedure and research tools

**Fig. 1:**
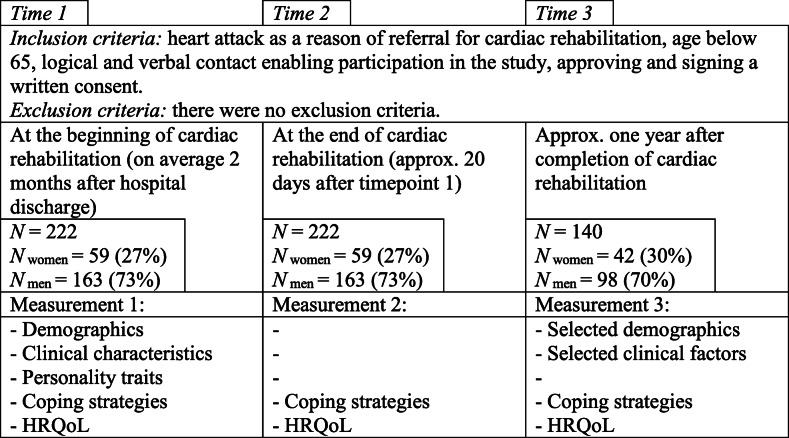
Procedure of the study

The study was conducted in natural settings, in a longitudinal design. It consisted of three stages described in Fig. 1. Timepoints 1 and 2 (T1 and T2) took place at the beginning and the end of the inpatient (stationary) cardiac rehabilitation at five rehabilitation centres in Poland. During the examination conducted by resident psychologists trained in the study protocol, subjects participated in the structured interview and filled in the set of paper–pencil questionnaires. Timepoint 3 (T3) was conducted by mail (including a return envelope) on average 11.62 months (standard deviation; SD = 1.9) after completion of rehabilitation; invitations were sent up to three times.

Part I of the Polish adaptation of the Nottingham Health Profile (NHP) [23, 24] was used to measure HRQoL and contained 38 statements about various ailments (yes/no answers) organised into six subscales. Due to correlations between subscales, factor analyses were performed, which revealed two higher-order factors. The symptoms factor (physical HRQoL) included the items on pain, immobility and lack of energy (Cronbach’s α values at T1 to T3 were 0.80, 0.80 and 0.87, respectively), and the psychosocial discomfort factor (mental HRQoL) included items on adverse emotional reactions, social isolation and sleeplessness (Cronbach’s α values = 0.70, 0.70 and 0.63, respectively). Together these higher-order factors explained 71, 72 and 75% of the variance in NHP scores at T1 to T3, respectively. Higher scores indicated a worse physical and mental HRQoL.

The Polish adaptation of the State-Trait Personality Inventory (STPI) [25] was used to assess anxiety, anger and curiosity traits. Each trait is measured using 10 items describing feelings and behaviour. Respondents indicate how typical each item is of them using a scale ranging from 1; ‘not at all’ to 4; ‘very much’. In our sample, Cronbach’s α reliability coefficients for the anxiety, anger and curiosity subscales were 0.84, 0.89 and 0.75, respectively. Higher scores indicate the higher intensity of traits.

The Polish adaptation of the Life Orientation Test-Revised (LOT-R) was used to measure optimism [26, 27]. It consists of 10 items describing beliefs about the future. Answers are given on a scale ranging from 0; ‘definitely does not apply to me’ to 4; ‘definitely applies to me’. Higher scores indicate greater optimism. The reported reliability coefficients of the original and Polish adaptations are 0.78 and 0.76 respectively; in our sample α = 0.56.

Coping strategies were measured using a modified version of the COPE questionnaire [27, 28] consisting of 46 statements organised into seven subscales. Answers were given on a scale ranging from 1; ‘I don’t usually do this at all’ to 4; ‘I usually do this a lot’. Cronbach’s α coefficients for the subscales ranged from 0.82 to 0.93, except for the resignation scale (0.60, 0.71 and 0.58 at successive timepoints, respectively). Higher scores indicate a higher frequency of strategies.

The study protocol was approved by the institutional Bioethics Committee. The patients were informed about the purpose and nature of the study and gave written consent for participation at all timepoints.

### Statistical analysis

The violation of normal distribution was verified with the Shapiro–Wilk test and Skewness and Kurtosis coefficients, independently for men and women. In cases where the variables were not normally distributed (HRQoL variables and selected coping variables) |SK| and |K| were less than 1 and did not exceed 1.7 and 2.1, respectively, except for substance use (the procedure of distribution normalisation did not bring a noticeable improvement).

Separate stepwise regressions were used to identify predictors of HRQoL at each timepoint. To maintain the proper relationship between the number of predictors and subjects (close to 15 observations per predictor) [29], the analyses were carried out separately for women and men in two steps. First, the statistically significant predictors (*p* < 0.05) were selected from each category, for each outcome variable and at each timepoint (4 × 2 × 3 = 24 models) for men and women. Next, these predictors were introduced blockwise into separate models for each outcome variable, at each timepoint (2 × 3 = 6 comprehensive models) for men and women. Only the results of the second step of the procedure are presented. The parameters for the final model, including all blocks of variables, are shown in Tables 3, 4 and 5, so some of the predictors from earlier blocks are statistically insignificant.

Post-hoc power analysis revealed that the statistical power of all the results for men was 0.80 or higher, with the alpha level set at 0.05. For women, the results for discomfort at T1 and T2, and for symptoms at T3, reached this value, therefore some results should be interpreted with caution.

## Results

### Characteristics of the group

T1 and T2 data were obtained from 222 MI survivors. Their sociodemographic and clinical characteristics are presented in Table 1. The men (*n* = 163) and women (*n* = 59) were similar with respect to most of the tested characteristics. At T1, men were more likely to be professionally active and to have been assigned to intensive cardiac training than women. At T3, the number of participants dropped to 140 participants (42 women and 98 men). Men and women did not differ in professional activity, as the number of men in work had fallen. The attrition analysis revealed that T1 and T3 samples were similar in respect of the majority of the sociomedical characteristics, except that the T3 participants had fewer comorbidities at baseline (χ^2^_(2, *n* = 222)_ = 10.31; *p* = 0.006) and were more likely to be engaged in low-level cardiac training (χ^2^_(2, *n* = 222)_ = 8.58; *p* = 0.02).

**Table 1:** Sociodemographic and clinical characteristics of the study group (*n* = 222)

*Characteristic*		*Differences based on gender*
Age [yrs]	*M* = 53.84; *SD* = 6.71 range 24–65	*t*(129) = − 1.66; *p* = .09
Marital status: married, n (%)	189 (85)	*χ*^2^ = 3.26=; *p* = .07
Employment prior to MI, n (%)		*χ*^2^ = 12.45; *p* < .001
Yes (M/W)	114 (71)/26 (44)	
Education, n (%)		*χ*^2^ = 3.37; *p* = .34
Vocational	105 (47)	
High school	86 (39)	
University-level	31 (14)	
Place of residence, n (%)		*χ*^2^ = 0.20; *p* = .90
Rural	52 (23)	
Town	96 (43)	
City	74 (33)	
Previous MI, n (%)		*χ*^2^ = 2.73, *p* = .07
First MI	189 (85)	
Type (STEMI/NSTEMI), n (%)		*χ*^2^ = 0.03, *p* = .48
STEMI	149 (67)	
Course, n (%)		*χ*^2^ = 2.01, *p* = .13
Uncomplicated MI	204 (92)	
PTCA, n (%)		*χ*^2^ = 2.59, *p* = .27
Yes	204 (92)	
Duration of hospitalization after MI, *M* ± *SD,* range [number of days]	7.33 ± 3.07 3–23	*χ*^2^ = 0.54, *p* = .46
Time from MI to the beginning of the study, n (%)		*χ*^2^ = 5.45, *p* = .14
Up to 30 days	122 (55)	
31–90 days	58 (26)	
More than 90 days	42 (19)	
Angina before MI, n (%)		*χ*^2^ = 6.06, *p* = .11
Negative	118 (53)	
Up to 1 month	38 (17)	
Up to 12 months	26 (12)	
Longer than 12 months	40 (18)	
Comorbidities, n (%)		*χ*^2^ = 1.04, *p* = .59
None	69 (31)	
Angina at T1, n (%)	164 (74)	*χ*^2^ = 1.36, *p* = .51
Beta blockers at T1, n (%)		*χ*^2^ = 1.12, *p* = .29
Yes	155 (70)	
Anxiolytics at T1, n (%)		*χ*^2^ = 0.88, *p* = .35
Yes	22 (10)	
Physical rehabilitation intensity at T1, n (%)		*χ*^2^ = 14.51, *p* = .001
High intensity M/W	52 (32)/4 (7)	
Moderate intensity M/W	96 (59)/48 (81)	
Breathing M/W	15 (9)/7 (12)	
Recurrent MI at T3 (*n* = 140), n (%)	6 (4)	*χ*^2^ = 2.85; *p* = .24
Rehospitalisation at T3 (*n* = 140), n (%)	30 (21)	*χ*^2^ = 2.28; *p* = .32
Employment at T3 (*n* = 140), n (%)	60 (43%)	χ^2^ = 2.76; *p* = .43

*MI* myocardial infarction, *STEMI* an ST-segment elevation myocardial infarction, *NSTEMI* a non-ST segment elevation myocardial infarction, *PTCA* percutaneous transluminal coronary angioplasty, *M* mean, *SD* standard deviation, *CHD* coronary heart disease, *M* men, *W* women

### Gender differences in personality, coping and HRQoL after MI

There were no gender differences in anger and optimism. Women reported higher trait anxiety and lower trait curiosity than men (Table 2).

**Table 2:** Gender differences in personality traits, coping strategies, and HRQoL dimensions

*Scale*		*Time 1 (n = 222)*			*Time 2 (n = 222)*			*Time 3 (n = 140)*		
		*M (SD)*	*min-max*	*F (p)*	*M (SD)*	*min-max*	*F (p)*	*M (SD)*	*min-max*	*F (p)*
Personality										
Anxiety trait	W	25.14 ± 4.90	14–35	18.78 (<.001)	–		–	–		–
	M	22.02 ± 4.67	10–36		–		–	–		–
Anger trait	W	25.07 ± 6.19	10–39	1.57 (.211)	–		–	–		–
	M	23.88 ± 6.27	12–37		–		–	–		–
Curiosity trait	W	29.9 ± 3.98	21–38	4.26 (.040)	–		–	–		–
	M	31.14 ± 3.95	18–40		–		–	–		–
Optimism	W	14.71 ± 3.77	6–24	1.19 (.277)	–		–	–		–
	M	15.36 ± 3.98	4–24		–		–	–		–
Coping strategies										
Reinterpretation	W	35.44 ± 6.08	22–44	4.74 (.031)	35.20 ± 5.25	23–46	5.91 (.016)	35.62 ± 6.76	15–48	97.00 (.327)
	M	33.18 ± 6.14	13–46		33.32 ± 5.84	14–47		34.47 ± 6.15	12–47	
Religion	W	10.97 ± 3.3	4–16	30.13 (<.001)	10.86 ± 3.34	4–16	24.11 (<.001)	9.86 ± 4.09	4–16	2.38 (.125)
	M	8.17 ± 3.38	4–16		8.31 ± 3.45	4–16		8.79 ± 3.62	4–16	
Humor	W	10.28 ± 3.5	5–18	10.1 (.002)	11 ± 3.22	6–17	4.38 (.038)	11.00 ± 3.22	6–20	1.17 (.281)
	M	12.04 ± 3.68	5–20		12.17 ± 3.84	5–20		12.17 ± 3.84	5–20	
Resignation	W	7.58 ± 2.17	4–13	2.12 (.147)	7.98 ± 2.24	4–13	4.47 (.036)	7.62 ± 2.4	4–12	3.76 (.054)
	M	7.12 ± 2.04	4–13		7.23 ± 2.29	4–13		6.87 ± 1.96	4–13	
Social support	W	18.97 ± 5.04	10–31	2.08 (.151)	19.88 ± 4.27	9–29	2.96 (.087)	18.45 ± 4.43	9–28	.04 (.842)
	M	17.93 ± 4.64	8–30		18.6 6 ± 4.8	8–30		18.3 ± 4.16	8–29	
Problem solving	W	23.69 ± 5.41	11–36	.30 (.584)	24.51 ± 4.5	14–33	1.05 (.307)	23.38 ± 5.42	11–34	.52 (.472)
	M	23.22 ± 5.78	9–35		23.7 ± 5.43	9–36		24.06 ± 4.97	9–33	
Substance use	W	4.88 ± 1.98	4–14	.96 (.330)	5.12 ± 2.25	4–12	1.31 (.255)	4.67 ± 1.9	4–12	1.25 (.214)
	M	5.18 ± 2	4–13		5.53 ± 2.44	4–12		5.11 ± 2.01	4–13	
HRQoL dimensions										
Physical (symptoms)		*Mdn; IQR*	*min-max*	*U (p)*	*Mdn; IQR*	*min-max*	*U (p)*	*Mdn; IQR*	*min-max*	*U (p)*
	W	3; 1–6	0–15	6121.5 (<.001)	2; 0–5	0–13	5777.5 (.015)	3; 0–6	0–16	2284.5 (.288)
	M	1; 0–4	0–14		0; 0–3.5	0–14		1; 0–5.75	0–12	
Mental (discomfort)	W	5; 3–7	0–17	6340.5 (<.001)	4; 1–7	0–14	6039 (.003)	3; 1–6	0–18	2378 (.142)
	M	2; 1–5	0–17		1; 0–4.5	0–17		2;0–6	0–16	

*M* mean, *SD* standard deviation, *Mdn* median, *U* the result of a Mann–Whitney U test, *W* women, *M* men, *IQR* interquartile range

At T1 and T2, women made more frequent use of reinterpretation and religion than men, whereas men made more use of humour than women. At T2, women also made more use of resignation. At T3, there were no gender differences in coping strategies (Table 2). At T1 and T2, women had a lower physical and mental HRQoL, but these differences had disappeared by T3 (Table 2).

### HRQoL predictors in females and males at the beginning of cardiac rehabilitation

Table 3 shows that at T1, none of the factors predicted women’s symptoms, although their discomfort was negatively predicted by education and trait curiosity, and positively predicted by trait anxiety and the use of resignation as a coping strategy. In men, symptom severity was negatively associated with being professionally active before MI, and positively associated with current angina and trait anger. Being professionally active and being optimistic were negatively associated with discomfort, whereas the use of anxiolytics, trait anxiety and trait anger were positively associated with discomfort.

**Table 3:** Predictors of hrqol at the beginning of cardiac rehabilitation in women and men – comprehensive models

	*Women*		*Men*	
*Predictors*	*Symptoms β(p)*	*Discomfort β(p)*	*Symptoms β(p)*	*Discomfort β(p)*
Demographics				
Education	–	−.21 (.03)	−.08 (ns)	–
Employment before MI	–	.02 (.ns)	−.18 (.02)	−.12 (.04)
Not included: age, marital status, place of residence	Δ*R*^2^ = 0	Δ*R*^2^ = .22 *p* = .001	Δ*R*^2^ = .10 *p* = .001	Δ*R*^2^ = .04 *p* = .009
Clinical characteristics				
Angina before MI	–	–	.12 (ns)	–
Angina at T1	–	–	.21 (.003)	.04 (ns)
Anxiolytics	–	–	.08 (.ns)	.15 (.01)
Not included: type of MI, PTCA, complications, number of MI, betablockers, length of hospitalization, comorbidity, time gap between MI and rehabilitation, intensity of physical training	Δ*R*^2^ = 0	Δ*R*^2^ = 0	Δ*R*^2^ = .13 *p* < .001	Δ*R*^2^ = .10 *p* < .001
Personality factors				
Curiosity-trait	–	−.33 (.003)	–	–
Anxiety-trait	–	.31 (.02)	–	.33 (.001)
Anger-trait	–	.20 (ns)	.31 (.001)	.23 (.008)
Optimism	–	–	–	−.19 (.003)
	Δ*R*^2^ = 0	Δ*R*^2^ = .36	Δ*R*^2^ = .09	Δ*R*^2^ = .37
		*p* < .01	*p* < .001	*p* < .001
Coping strategies				
Resignation at T1	–	.19 (.04)	–	−.01 (.ns)
Religion at T1	–	–	.10 (.ns)	.09 (.ns)
Substance use at T1	–	–	–	.06 (ns)
Not included: reinterpretation, humor, social support, problem solving	Δ*R*^2^ = 0	Δ*R*^2^ = .03 *p* = .04	ΔR^2^ = .01 *p* = ns	Δ*R*^2^ = .01 *p* = ns
Total	adj*R*^2^ = 0	adj*R*^2^ = .57	adj*R*^2^ = .30	adj*R*^2^ = .49

If ‘-‘, the predictor was not included in the given analysis; not included, not entered into the comprehensive model

*ns* not significant

### HRQoL predictors in females and males at the end of cardiac rehabilitation

Table 4 shows that at T2, none of the factors investigated predicted women’s symptoms, although there were marginal negative relationships between symptoms and substance use, and positive with resignation. Education continued to be a negative predictor of discomfort, whilst the use of anxiolytics and resignation at T1 were positive predictors of discomfort. In men, the predictors of symptoms were, as at T1, pre-MI employment, current angina and trait anger; there was also a delayed effect of duration of angina before MI. The predictors of discomfort in men were the same as at T1, except that the effect of professional activity had disappeared.

**Table 4:** Predictors of hrqol at the end of cardiac rehabilitation in women and men – comprehensive models

	*Women*		*Men*	
*Predictors*	*Symptoms* *β(p)*	*Discomfort* *β(p)*	*Symptoms* *β(p)*	*Discomfort* *β(p)*
Demographics				
Education	–	−.27 (.04)	–	−.08 (ns)
Employment before MI	–	–	−.17 (.02)	−.08 (ns)
Not included: age, marital status, place of residence	Δ*R*^2^ = 0	Δ*R*^2^ = .10 *p* = .02	Δ*R*^2^ = .08 *p* < .001	Δ*R*^2^ = .08 *p* = .002
Clinical characteristics				
Angina before MI	–	–	.19 (.009)	–
comorbidity	–	–	.09 (ns)	–
Angina at T1	–	–	.31 (.001)	0.07 (ns)
Anxiolytics	–	.25 (.04)	.03 (ns)	.21 (.001)
Intensity of physical training			−.10 (ns)	–
Not included: type of MI, complications, PTCA, number of MI, betablockers, length of hospitalization, time gap between MI and rehabilitation	Δ*R*^2^ = 0	Δ*R*^2^ = .06 *p* = .*06*	Δ*R*^2^ = .24 *p* < .001	Δ*R*^2^ = .12 *p* < .001
Personality factors				
Curiosity-trait	–	–	–	–
Anxiety-trait	–	–	–	.23 (.02)
Anger-trait	–	–	.26 (.001)	.17 (.*06*)
Optimism	–	–	–	−.23 (.001)
	Δ*R*^2^ = 0	Δ*R*^2^ = 0	ΔR^2^ = .07	ΔR^2^ = .26
			*p* < .001	*p* < .001
Coping strategies				
Resignation at T1	–	.38 (.008)	–	–
Problem solving at T1	−.18 (ns)			–
Religion at T1	–	–	–	.18 (.ns)
Substance use at T1	–	–	–	.08 (ns)
Substance use at T2	−.26 (.08)	–	–	–
Resignation at T2	.29 (.051)	−.10 (ns)	–	–
Religion at T2	–	–	–	−.01 (ns)
Problem solving T2	–	–	−.08 (ns)	–
Not included: T1 and T2 reinterpretation, T1 and T2 humor, T1 and T2 social support	Δ*R*^2^ = .12 *p* = .07	Δ*R*^2^ = .12 *p* = .02	ΔR^2^ = .006 *p* = ns	ΔR^2^ = .03 *p* = ns
Total	adj*R*^2^ = .07	adj*R*^2^ = .22	adj*R*^2^ = .36	adj*R*^2^ = .45

If ‘-‘, the predictor was not entered into a block; not included, not entered into the comprehensive model

*ns* not significant

### HRQoL predictors in females and males one year after cardiac rehabilitation

Table 5 shows that at T3, the only predictor of women’s symptoms was angina at T3, although there was also a marginal association with professional activity at T3. Discomfort was positively predicted by use of beta-blockers at T1, angina at T3 and substance use at T2. In men, the strongest predictor of symptoms was angina at T3. The use of problem-solving at T1 was a negative predictor of discomfort at T3 (a delayed effect), whereas resignation at T3 was a positive predictor of discomfort.

**Table 5:** Redictors of hrqol one year after completion of cardiac rehabilitation in women and men – comprehensive models

	*Women*		*Men*	
*Predictors*	*Symptoms* *β(p)*	*Discomfort* *β(p)*	*Symptoms* *β(p)*	*Discomfort* *β(p)*
Demographics				
Marital status	.09 (ns)	–	–	–
Education	–	–	−.13 (ns)	−.16 (ns)
Employment before MI	–	–	−.16 (.07)	–
Employment at T3	−.25 (.06)		–	−.13 (ns)
Not included: age, place of residence	Δ*R*^2^ = .22 *p* = .007	Δ*R*^2^ = 0	Δ*R*^2^ = .17 *p* < .001	Δ*R*^2^ = .12 *p* = .002
Clinical characteristics				
Betablockers	–	.29 (.04)	–	–
Comorbidity	.15 (ns)	–	–	–
Angina at T1	–	.22 (ns)	–	–
Anxiolytics	–	–	.11 (ns)	.10 (ns)
Rehospitalisation		–	.17 (*.*07)	–
Angina at T3	.49 (.001)	.36 (.01)	.35 (.001)	–
Not included: type of MI, PTCA, complications, number of MI, length of hospitalization, time gap between MI and rehabilitation, angina before MI, intensity of physical training, new MI	Δ*R*^2^ = .27 *p* < .001	Δ*R*^2^ = .28 *p* = .006	Δ*R*^2^ = .21 *p* < .001	Δ*R*^2^ = .04 *p* = .04
Personality factors				
Anxiety-trait	–	–	–	–
Optimism	–	–	−.06 (ns)	−.08 (ns)
Not included: anger-trait, curiosity-trait	Δ*R*^2^ = 0	Δ*R*^2^ = 0	Δ*R*^2^ = .02 *p* = ns	Δ*R*^2^ = .02 *p* = ns
Coping strategies				
Social suport at T1	–	–	–	.18 (ns)
Resignation at T1	–	–	.10 (ns)	–
Problem solving at T1	–	–	−.17 (ns)	−.30 (.02)
Substance use at T2	–	.31 (.03)	–	–
Resignation at T2	–	–	.06 (ns)	.11 (ns)
Humour at T2	–	–	−.16 (*.08*)	–
Problem solving at T2	–	–	.06 (ns)	–
Resignation at T3	–	–	.09 (ns)	.22 (.03)
Not included: T1, T2, T3 reinterpretation, T1, T2, T3 religion, T1 and T3 humor, T2 and T3 social support, T1 and T3 substance use, T3 problem solving	Δ*R*^2^ = 0	Δ*R*^2^ = .09 *p* = .03	Δ*R*^2^ = .05 *p* = ns	Δ*R*^2^ = .11 *p* = .01
Total	adj*R*^2^ = .44	adj*R*^2^ = .30	adj*R*^2^ = .37	adj*R*^2^ = .23

If ‘-‘, the predictor was not entered into a block; not included, not entered into the comprehensive model

*ns* not significant

### HRQoL predictors after controlling for HRQoL at earlier timepoints

Given that there were gender differences in HRQoL at T1 and T2, we checked whether predictors of T2 and T3 HRQoL remained significant after controlling for HRQoL at the previous timepoints.

Using this approach there were two predictors of women’s symptoms at T2: substance use at T2 (β = − 0.24, *p* = 0.01) and resignation at T2 (β = 0.20, *p* = 0.04). Education, use of anxiolytics and resignation at T1 no longer predicted T2 discomfort. Symptoms and discomfort at T1 explained an additional 59 and 54% of the variance in T2 symptoms and discomfort, respectively (*p* < 0.001 for each). In men, T2 symptoms were still predicted by pre-MI angina and angina at T1, but pre-MI employment and trait anger were no longer significant predictors. Use of anxiolytics and optimism still predicted T2 discomfort, but trait anxiety did not. Symptoms and discomfort at T1 explained the additional 51 and 50% of the variance in T2 symptoms and discomfort, respectively (*p* < 0.001 for each).

In women, T3 outcomes were very similar to those reported in the previous section. Adding symptoms at T1 and T2 did not increase significantly the amount of variance in T3 symptoms (6%, *p* = 0.29; symptoms at T1 β = 0.33, *p* = 0.09; symptoms at T2 β = 0.08, *p* = 0.69). Substance use at T2 and angina at T3 still predicted women’s T3 discomfort, but use of beta-blockers did not. Adding discomfort at T1 and T2 did not increase significantly the amount of variance in T3 discomfort (10%, *p* = 0.08; discomfort at T1 β = 0.03, *p* = 0.93; discomfort at T2 β = 0.03, *p* = 0.39). In men T3 outcomes were also very similar to those reported previously. Adding symptoms at T1 and T2 increased the amount of variance in T3 symptoms (14%, *p* < 0.001), however symptoms at T1 and T2 were not significant predictors (β = 0.33, *p* = 0.09 and β = 0.08, *p* = 0.69, respectively). Problem-solving at T1 and resignation at T3 still predicted men’s T3 discomfort. Adding discomfort at T1 and T2 increased the amount of variance in T3 discomfort (11%, *p* = 0.002), however discomfort at T1 and T2 were not significant predictors (β = 0.09, *p* = 0.48 and β = 0.23, *p* = 0.07, respectively).

## Discussion

Our finding that women had a lower HRQoL than men at T1 and T2 is consistent with other research on short-term recovery from MI [9, 12, 21]. It related to both components of HRQoL, but many studies indicate that the effect depends on the HRQoL dimension [21]. Also, like other studies of longer-term recovery [6–8], the difference in HRQoL was no longer present 1 year after MI.

The factors predicting HRQoL over one year after MI found in females were different than those found in males. We did not find predictors of women’s physical HRQoL during short-term recovery, and the reasons for this remain unclear [30]. As in a previous study [31], neither age, comorbidities nor characteristics of MI predicted HRQoL, however, unlike the earlier study, [31] neither employment nor marital status were predictors in women. When adopting a longitudinal approach, some predictors which were previously insignificant (substance use and resignation) could predict symptoms; this can be interpreted as a suppression effect [32] and confirm the complexity of HRQoL correlates. Generally, longitudinal analyses reveal some changes in the T2 outcomes in both genders, with baseline HRQoL emerging as the strongest predictor of HRQoL three weeks later. Another study has also reported similar effect over 6 months [31], although it did not refer to the results at the one-year follow-up when we controlled for HRQoL at previous stages (when genders differed in HRQoL). The possibility of comparing outcomes from the cross-sectional and longitudinal analyses is an added value of our study.

One year after MI, nearly 20% of survivors still experience angina [33], and it can reduce patients’ HRQoL at one-year follow-up and later [34–37]. In our study, angina in men predicted symptoms at all timepoints, whereas, for women, the effect appeared only after a year; this can be partially explained by women being more dependent than men on external cues in defining their symptoms [21].

Our results confirmed that pharmacological treatment also plays a vital role in HRQoL. Arendarczyk [38] found that use of beta-blockers two years after MI was positively associated with HRQoL, whereas another study found that they exacerbated the functional decline in older nursing homes residents with cognitive impairment [39]. We found that in women, the delayed and unbeneficial effect of taking beta-blockers on discomfort may persist for around a year. Also, the use of anxiolytics, due to anxiety disturbance after MI, may affect HRQoL in both men and women [5]. Another aspect of post-MI distress is depression, which was not included in the study; it has been estimated that depressive symptoms are from twice to three times more prevalent in patients with coronary artery disease and more frequent in women [40]. Some findings suggest that females and males may differ in the impact of depression on HRQoL [21].

It is interesting that at early timepoints, men’s employment status predicted both aspects of their HRQoL, whereas in women education predicted discomfort. The positive effect of education on HRQoL has been observed in several studies of men [5], but not in research on women [31]. The positive effect of employment in men may reflect the fact that, in our sample, the men were more likely to have been professionally active before MI than the women [41]. Being employed before MI increased the chance of returning to professional activity, which has been shown to reduce emotional distress [42]. We observed a fall in the proportion of men employed over time, which may have contributed to the deterioration in men’s HRQoL. Working less, or not working one year after MI, have been related to depression and lower health status [43].

Personality only predicted HRQoL at early timepoints. This is intriguing—why mental HRQoL was predicted by openness to novelty (trait curiosity) in women, but expectancy of a good future (optimism) in men, especially as in our sample, trait curiosity was lower in women, and there was no gender difference in optimism. Further research is needed to understand this issue, as it may help to better tailor secondary prevention interventions. Similar suggestions concern the negative impact of trait anger in men; anger, hostility and aggression have long been recognised as risk factors for the onset and progression of coronary heart disease [44]. It has also been shown that there is a strong negative association between anger and mental HRQoL when controlling for effects of gender, age and functional status [45]. However, a study of women with coronary heart disease found that trait anger was not related to any aspect of HRQoL [31]. Anxiety traits seem to play a similarly negative role in both genders, which is in line with other studies [17] showing the negative relationship between anxiety and HRQoL in cardiac patients during long-term recovery [46]. Particular attention should be paid by health care professionals to these highly anxious patients, regardless of gender.

Ways of coping, although seen as important contributors to HRQoL [47], are not often investigated in this context [17, 31]. It is assumed that gender differences in coping, for example, women’s more frequent use of disengagement strategies [20, 48] and their tendency to use a greater variety of coping strategies [21], may account for gender differences in HRQoL. However, Brink [19] observed no gender differences in using coping strategies after MI. We observed that resignation predicted HRQoL at early stages in women and in men at one-year follow-up. However, in our sample, there was no gender difference in the dynamics of the use of this strategy [49], although women generally made more use of resignation than men. This shows that even when there is no gender difference in the use of coping strategies, they can relate to HRQoL in a different way in females and in males.

Another important observation relates to delayed effects. It appears that in men, but not women, applying problem-solving strategies during early cardiac rehabilitation has a delayed effect on mental HRQoL one year later. Again, there was no gender difference in the dynamics of this strategy [49]. Unfortunately, the delayed positive effect of problem-solving was counterbalanced by a negative effect of resignation. In women, the directions of the delayed effects of substance use changed depending on time (there were no gender or time differences in the use of this strategy) [49]. Some interrelations between HRQoL dimensions are also possible, as substance use reduced symptoms at the end of rehabilitation (in longitudinal analysis), and increased discomfort at one-year follow-up. This suggests that using prescribed or over-the-counter medication to help one to forget about problems and to console oneself, produces a short-term reduction in physical symptoms, but increases psychosocial discomfort in the long term. Probably, it contributes to unrealistic expectations or is associated with the unforeseen deterioration in physical condition. These speculations and possible indirect relationships between determinants need to be verified in further research.

### Limitations of the study

Due to the exploratory character of the study, caution is needed in drawing final conclusions, especially in relation to the results for women. The constraints resulting from the relatively small sample size relative to the number of predictors did not allow us to perform full verification of the moderation effects. In an attempt to restrict the number of predictors in the model to an acceptable number, we adopted a two-step selection procedure. Women constituted approximately 30% of the sample, but the proportion of women was very similar to that in other mixed-gender samples [6, 9, 12]. Although the absolute number of women was relatively small, in the majority of analyses, we met the satisfactory statistical power. A bigger sample size with similar numbers in groups is recommended in any future study.

Our indicator of HRQoL was a specially developed transformation of NHP scores, which may not be strictly comparable with other HRQoL measures used in cardiac studies, although the content is similar. However, this generic tool makes it possible to draw comparisons with other clinical groups. The groups were homogeneous with respect to almost all sociomedical characteristics, with the exceptions of employment status and intensity of rehabilitation training; this may be partially responsible for a small number of the effects we observed. We did not measure some probable determinants of HRQoL, for example, the level of depression [12], post-menopausal symptoms [13] and health-related behaviours [5]. Not taking these factors into account in explanatory models can lead to misleading conclusions. We recommend that future studies use a broader or different selection of predictors. As for the future direction of the study, gender differences in the impact of individual predictors on the HRQoL trajectory should be taken into account.

## Conclusions

Factors shaping HRQoL after MI were different in females and males, and they partially changed over the recovery process. Substantially fewer factors predicted symptoms in women than in men. There were noticeable differences in the psychosocial predictors of HRQoL, but trait anxiety was a predictor in both men and women. Further studies with a broader or different selection of predictors are needed and a greater emphasis should be placed on including women in research.

## Data Availability

The datasets used during the current study are available from the corresponding author on reasonable request.

## Abbreviations

HRQoL: Health-related quality of life
MI: Myocardial infarction
NHP: The Nottingham Health Profile
STPI: The State-Trait Personality Inventory
LOT-R: The Life Orientation Test-Revised
T1: Timepoint 1
T2: Timepoint 2
T3: Timepoint 3
